# The benefit of rhythm-based interventions for individuals with autism spectrum disorder: a systematic review and meta-analysis with random controlled trials

**DOI:** 10.3389/fpsyt.2024.1436170

**Published:** 2024-09-27

**Authors:** Xiaofen Ding, Jinlong Wu, Dong Li, Zexi Liu

**Affiliations:** ^1^ College of Physical Education, Hunan First Normal University, Changsha, China; ^2^ College of Physical Education, Southwest University, Chongqing, China; ^3^ Department of International Culture Education, Chodang University, Muan, Republic of Korea; ^4^ College of Art, Chonbuk National University, Jeonju, Republic of Korea

**Keywords:** rhythm, ASD, social skills, communication, emotion, empathy, meta

## Abstract

**Objective:**

Individuals with autism spectrum disorder (ASD) exhibit impaired behavior synchronization, which is associated with social deficits. Numerous studies have demonstrated that rhythm-based interventions can effectively mitigate social deficits by promoting behavioral synchronization in individuals with ASD. Therefore, a review of the current literature is warranted in this field. The objectives of this review were to explore the effects of rhythm-based interventions on overall social skills and to study the differences in the effects of rhythm-based interventions on specific social skills.

**Method:**

The databases PubMed, Web of Science, Scopus, and Psycinfo were systematically explored until March 2024. A total of eleven research studies, encompassing 408 participants diagnosed with ASD, were incorporated into the meta-analysis. Effect sizes (Hedges’ g) were computed for each comparison and amalgamated using random-effects models to evaluate the social skills of individuals with ASD. The methodological quality of each study was evaluated using the Physiotherapy Evidence Database scale(PEDro).

**Results:**

Overall, some valuable observations were made. Rhythm-based interventions had a medium effect on the overall social skills for ASD (Hedges’s=0.681; 95%CI[0.075 to 1.286], P  < 0.05). Regarding domain-specific social skills, rhythm-based interventions had a large effect on social interaction (g = 1.299,95% CI [0.508 to 2.091]), a small effect on communication (g = 0.383, 95% CI [0.033 to 0.733], P  < 0.05), and a large effect on emotion (g = 1.752, 95% CI [0.294 to 3.210], P  < 0.05). However, we found a favorable but non-significant effect (g = 0.125, 95% CI [-0.039 to 0.289], P  >  0.05) of rhythm-based interventions on empathy. All study qualities were high (score≥6) using the Physiotherapy Evidence Database (PEDro) scale assessment.

**Conclusion:**

This result indicates the importance of rhythm in the clinical rehabilitation of individuals with ASD. We suggest adding appropriate rhythmic elements to clinical interventions, particularly for individuals with ASD who are less socially competent.

## 1 Introduction

Autism spectrum disorder (ASD), a pervasive neurodevelopmental disorder, is distinguished by challenges in social communication and interaction, in addition to repetitive behaviors, interests, and activities that may persist throughout one’s life (1). The prevalence of ASD has increased in recent decades, with 1 in 59 children in the United States and over 600,000 people in the United Kingdom, equivalent to a population prevalence of ∼1% (2). Continuing deficits in social communication and social development can be highly problematic for individuals diagnosed with ASD (3). These difficulties can impact a person’s ability to initiate social interactions, engage in reciprocal conversation, and maintain eye contact and joint attention (4, 5). Such challenges can lead to difficulties in forming and maintaining relationships, ultimately resulting in feelings of social isolation, loneliness, and a diminished quality of life (6).

Researchers has been focused on improving communication and social skills in children with ASD through evidence-based therapeutic interventions (7). Recent studies have been found rhythm-based interventions show several positive effects that enhance communication skills and develop social interactions in individuals with ASD (8–10). These rhythm-based interventions typically encompass a strong, consistent, and recurrent pattern of movement or sound (11–13). Strong rhythmic patterns can may trigger behavioral, neurological, and physiological rhythm changes in individuals with ASD that promote social connections (14). First, some studies found that after the rhythm-based intervention, the synchronization of social movement will be significantly improved for individuals with ASD (15, 16). Second, brain imaging studies have confirmed that rhythmic experiences is closely related to neural synchronization and play a key role in developing basic trust and feelings of connection with others (17, 18). A studies found compared to key-press task alone, rhythmic keystrokes can improve interbrain synchronization between children with ASD and their parents (19). Three, one study showed the act of dancing to music has been proven to trigger physiological synchronization, specifically in terms of heart rate intervals (20). Therefore, incorporating aspects of rhythm cues into interventions for patients with ASD holds great promise mainly because of the increasing emphasis on sensorimotor function in interventions for this population.

Rhythmic-based interventions (such as music and dance therapy) have increasingly emerged as promising compensation methods that can positively affect negative symptoms in individuals with ASD. A meta-analysis (21) showed that dance practice significantly alleviated the overall symptoms of ASD (standardized mean difference (SMD)=−1.48, 95%confidence interval(CI)[−2.55, −0.42], P =0.006, I^2^ = 75%) and improved social interaction(SMD=0.88, 95%CI [0.46 to1.30], P <0.0001, I2 = 0%), but had no significant effect on empathy (SMD=0.09, 95%CI [−0.25 to 0.42], P =0.61, I^2^ = 2%). Another recent meta-analysis (22) showed that music therapy was associated with a significant increase in social reactions among children with ASD (SMD=0.24, 95%CI [0.03, 0.46], I^2^ = 0%, P=0.03). However, music therapy did not elicit a significant increase in symptom severity (SMD=0.17, 95%CI[−0.04 to 0.38], I^2 =^ 0%, P=0.12), social adaptive behavior (SMD=0.02, 95%CI[−0.44, 0.48], I^2^ = 0%, P=0.93), or speech (SMD=0.04, 95%CI[−0.39 to 0.47], I^2^ = 0%, P=0.86) in children with ASD.

Nonetheless, we have identified three gaps in the literature. First, previous studies rarely focused on the importance of rhythmic effects on social skills in individuals with ASD. Second, few reviews have distinguished between specific types of social skills (i.e., communication, social interaction, or emotion). It is difficult to identify the effects of rhythmic-based interventions on specific types of social skills among individuals with ASD. Furthermore, past reviews and meta-analyses had limitations including small sample sizes, absence of a control group, and study designs such as case studies or single-subject research lacking outcome measures.

Considering the crucial role of rhythm-based intervention for individuals with ASD in enhancing social skills. To better understand the effects of rhythmic interventions on social skills, a review of the current literature is warranted. The aim of the present meta-analysis, therefore, is to quantitatively evaluate the most recent studies on the effects of rhythm-based interventions on social skills of ASD individuals. It helps clinicians and rehabilitators design better intervention programs with key treatment factors for individuals with ASD. Consequently, the objectives of this review were to explore the effect of rhythm-based interventions on overall social skills and to study the effect of rhythm-based interventions on specific social skills.

## 2 Methods

The meta-analysis complied with the Preferred Reporting Items for Systematic Reviews and Meta-analyses(PRISMA) reporting checklist (23). Detailed search terms are shown in **Supplementary Table 1**.

### 2.1 Search strategy

We systematically searched the literature on the effect of rhythm-based interventions on social interaction skills for patients with ASD in PubMed, Web of Science, Scopus, and Psycinfo from the beginning of database construction of each database until March 2024. Our search strategy combined terms related to rhythm-based interventions (such as music, dance, or drums) and ASD. Additionally, we conducted a thorough manual search of the reference lists in all studies that were included, along with a comprehensive review to uncover additional pertinent articles (4, 21, 22, 24–27). Detailed search terms are shown in **Supplementary Table 2**.

### 2.2 Eligibility criteria

The quantitative synthesis for meta-analysis included only randomized controlled trials (RCTs) published in peer-reviewed English journals and reporting sufficient statistical details. Studies that did not involve comparison groups or did not provide comparison results between groups were not considered. Observational studies and other types of non-RCTs, such as cross-sectional, case-control, and cohort studies, as well as reviews, reports, papers, and policy documents, were excluded from the analysis.

The study focused on individuals diagnosed with ASD based on the criteria in the DSM (The Diagnostic and Statistical Manual of Mental Disorders) or other standardized diagnostic measures. There were no age restrictions. Excluded were studies that included participants with various disabilities that made it difficult to separate data relevant to children and adolescents with ASD. The main inclusion was any form of intervention that included rhythmic experiences, such as singing, playing the piano, drumming, etc., to the exclusion of other types of auditory stimuli that did not include rhythm. The studies that were not considered auditory interventions. Studies have measured social skill outcomes using validated instruments, such as communication questionnaires, emphasizing social skills.

### 2.3 Study selection and data extraction

Records that align with the specified search criteria will be imported into Endnote 20 software (Clarivate Inc., Philadelphia, PA, USA) for deduplication purposes. Two reviewers independently undertook the multi-step search process, evaluating studies based on titles, abstracts, and full texts to carry out an initial review. Any discrepancies between the reviewers were deliberated upon until consensus was achieved. If no agreement could be reached, a third reviewer intervened, making a conclusive decision after consultation with the original two reviewers. The agreement between the two reviewers during abstract and full-text screening was quantified using kappa values, in accordance with prior guidelines. The specific reasons for excluding full-text studies are detailed in **Supplementary Table 3**.

A standardized data extraction form was developed to meticulously collect and document the characteristics of each study. To ensure the accuracy and reliability of the data extraction process, it was performed independently by two reviewers. In instances where discrepancies arose, a third reviewer was involved to independently perform data extraction for resolution. The data collected encompassed a variety of crucial elements, including bibliographic details such as the author and year of publication, country/regions, participant characteristics like diagnostic criteria, sample age, sex, and sample size, as well as detailed descriptions of the intervention components, which covered the design, intensity, frequency, and length of the intervention, along with its setting. Information on the control group, outcome measures, and any relevant data was also meticulously documented. When certain relevant data were not available in the original papers, email inquiries were made to the corresponding authors to obtain the missing information. In cases where there was no response or the data could not be provided, the missing information was marked as “not applicable” (N.A.). Additionally, the ImageJ processing program (Version 1.50i, https://imagej.nih.gov/ij/) was employed to calculate the pixel value statistics for the defined selections and to extract numerical data from the figures.

### 2.4 Quality assessment

Two reviewers independently assessed the methodological quality of each study included in the analysis using the PEDro scale, which comprises 11 criteria related to eligibility, randomization, allocation, blinding of subjects and experimenters, intention-to-treat analysis, between-group comparisons, and outcome measures (28). The scores on the PEDro scale range from 0 to 11, with a median score of 6. Studies were categorized as excellent (9–10 points), good (6–8 points), fair (4–5 points), or poor (less than 4 points) quality (29). Any discrepancies in quality ratings between reviewers were resolved through discussion until a consensus was reached, with a third researcher intervening if necessary to make the final decision.

### 2.5 Data analysis

A meta-analysis was carried out to investigate the impact of exercise interventions on EFs. SMDs with 95% CIs were calculated for each study when utilizing different tools to assess outcome variables. The SMDs were determined and weighted based on the inverse variance of the study, taking into account sample sizes, outcomes, and cognitive measures. A random-effects model was applied to calculate potential effect sizes that were distributed heterogeneously, utilizing sampling error and between-study variance for effect size estimation (30).

Hedges’ served as the effect size indicator to address potential overestimation when fewer than 20 studies were included (31). The magnitude of Hedges’ values was categorized as small (Hedges’ g = 0.2–0.5), moderate (Hedges’ g = 0.5–0.8), and large effect (Hedges’ g > 0.8) (32). Statistical heterogeneity was assessed using I^2^ and the p-value for Q statistics, with I^2^ values representing low (≤ 25%), moderate (50%), or high (≥ 75%) levels of heterogeneity. Due to the inclusion of fewer than ten studies in each analysis, publication bias was not explored (33). The analysis was conducted utilizing the Comprehensive Meta-analysis version 3 software (Biostat, USA) (34).

## 3 Results

### 3.1 Study identification

**Figure 1:**
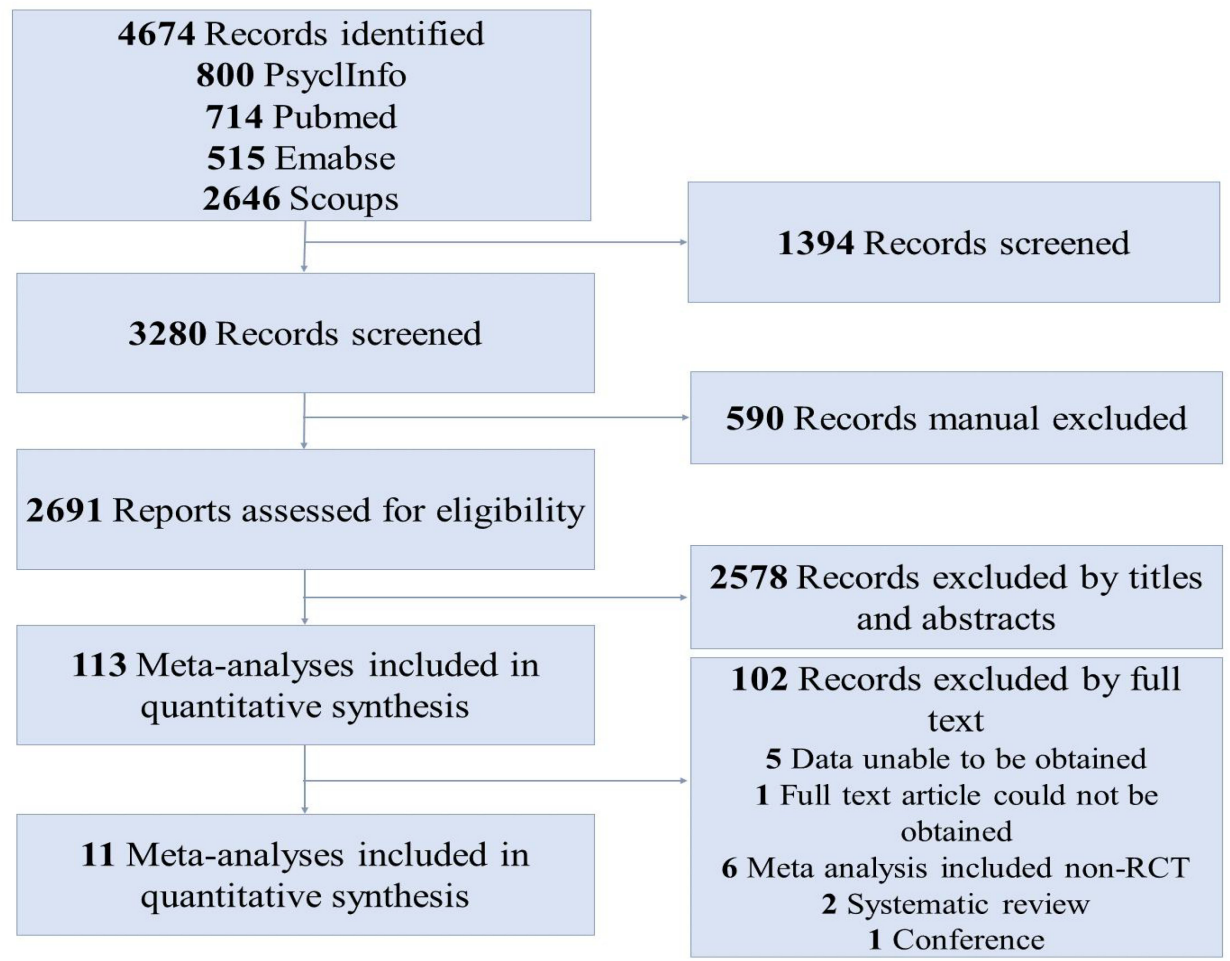
Flow diagram of the article screening process.

**Figure 1** shows the detailed article screening process. The initial search identified 4674 records from electronic databases using our literature search strategy (PubMed, n=714; Embase, n=515; PsycInfo, n=800; Scopus, n=2646). After deleting 1394 duplicate records using Endnote software, 3280 were screened. The manual deleted 590 duplicate records by a reviewer, and 2691 records were included. Of which, 2578 did not meet the inclusion criteria of this study based on their titles and abstracts. Finally, the full-text articles of 33 studies were screened; 22 were excluded, and 11 (35–45) were included in the present meta-analysis (**Figure 1**).

### 3.2 Study characteristics

The characteristics of each study are summarized in **Table 1**. 11 (35–45) studies were included in this meta-analysis published from 2014 to 2023, suggesting that rhythm-based interventions for social skills in individuals with ASD have been explored constantly in this field. Of the included interventions, four (35, 39, 40, 42) were conducted in the Germany, two (41, 44) were conducted in USA. The remaining intervention studies were conducted in Iran (38), Canada (43), India (36), Brazil (45), and Romani (37).

**Table 1:** Study characteristics meeting inclusion criteria.

Study	Country/regions	Diagnostic criterion	Sample age(years)	Male/female	Sample Size	Intervention	Setting	Control	Outcome measure
LaGasse (2014) (41)	USA	CARS2	Mean:15.78 Range:6-9	17/4	IG: 9 CG: 8	Music-based social skill training Sessions 50 min Frequency:2 days/week Duration: 5 weeks Total time:	Group`	Social skill training	SRS ATEC
Ghasemtabar et al. (2015) (38)	Iran	CARS	IG:8.96 ± 1.36 CG:9.23 ± 1.54	IG:7/6 CG:7/7	IG: 13 CG: 14	Music training Sessions: 30min Frequency:2 days/week Duration: 1.71 weeks Total time:	NA	Waitlis	SSRS
Koch et al. (2015) (39)	Germany	ICD-10	Mean:22.0 Range:16-47	NR	IG: 16 CG: 15	Dance movement therapy Sessions: 60min Frequency:1 days/week Duration: 7 weeks Total time:	Group	Waitlis	**Psychological well-being**: HIS **Body awareness:** Subscale body awareness of QMT Empathy: EES **Social skills** subscale social skills of the FBT
Srinivasan et al. (2015) (44)	USA	ADOS-2	IG: 7.88 ± 2.56 CG: 7.36 ± 2.02	IG:10/2 CG:11/1	IG: 12 CG: 12	Rhythm training Sessions: 45 min Frequency:4 days/week Duration: 8 weeks Total time:	Individually	Tabletop activities	sensory, negative, and stereotyped behaviors; Affective states
Koehne et al. (2016) (16)	Germany	DSM -5 ICD-10	IG: 33.5 ± 9.1 CG: 32.0 ± 9.1	IG:18/9 CG:14/10	IG: 27 CG: 24	Dance/movement training Rhythm training Sessions: 90 min Frequency:10 days/3 months Total time:	Group	Tradition movement training	Empathy:MET Interpersonal: IRI imitation/synchronization: ASIM
Mastrominico et al. (2018) (42)	Germany	ICD-10	Mean:22.5 Range:14-52	IG:27/8 CG:17/4	IG: 34 CG: 19	Dance movement therapy Sessions 60 min Frequency: 1 days/week Duration: 10 weeks Total time:	Group	Waitlis	Empathy: CEEQ; IRI
Sharda et al. (2018) (43)	Canada	DSM-4	IG: 10.30 ± 1.91 CG: 10.20 ± 1.87	IG:21/5 CG:22/3	IG:26 CG:25	Music Sessions:45 min Frequency: 1 days/week Duration:8–12 weeks	Individually	Non-music control intervention	Social communication: CCC-2 symptom severity: SRS-2 receptive vocabulary: PPVT-4 receptive vocabulary: VABS-MB FQoL
Bharathi et al(2019) (8)	India	DSM-5	IG: 10.30 ± 1.91 CG: 10.20 ± 1.87	IG:21/5 CG:22/3	IG:26 CG:25	Music and dance Sessions:45 min Frequency: 1 days/week Duration:8–12 weeks	Individually	Non-music control intervention	Social communication: CCC-2 symptom severity: SRS-2 receptive vocabulary: PPVT-4 receptive vocabulary: VABS-MB FQoL
Bergmann(2021) (35)	Berlin, Germany	Clinical doctor	9.5 ± 2.27	IG:4/0 CG:4/0	IG:4 CG:4	Dance/movement therapies Sessions 90 min Duration: 16 times Total time:	Group	Waitlis	**Social skills** SRS autcom social **Emotional skill:** Autcom emotional **Challenging behavior** ABC MOAS **Quality of life** POS
Teixeira-Machado et al. (2022) (45)	Brazil	Clinical doctor	IG: 10.41 ± 2.24 CG: 10.00 ± 2.03	IG:14/3 CG:15/4	IG: 17 CG:19	Dance/movement therapies Sessions: 40min Frequency: 1 days/week Duration: 24weeks	Group	Standard care	ABC ASQ CARS FIM
Dănciulescu et al. (2023) (37)	Bucharest, Romania	Clinical doctor	IG: 4.37 ± 1.19 CG: 4.80 ± 1.35	IG:26/4 CG:25/5	IG: 30 CG:30	Piano therapies Sessions: 50min Frequency: 2 days/week Duration: 4 months	Individually	Standard care	PEP-3; Examiner Report Form; Caregiver Report Form

CARS2, Childhood Autism Rating Scale, Second Edition; ICD-10, International Classification of Diseases 10th Revision; ADOS-2, Autism Diagnostic Observation Schedule, Second Edition; DSM -5, Diagnostic and Statistical Manual of Mental Disorders, Fifth Edition; DSM-4, Diagnostic and Statistical Manual of Mental Disorders, Fourth Edition; IG, Intervention Group; CG, Control Group; NA, Not Applicable; SRS, Social Responsiveness Scale; ATEC, Autism Treatment Evaluation Checklist; SSRS, Social Skills Rating System; QMT, Quran Memorization Tool; EES, Emotional Expression Scale; FBT, Fragebogen fuer bewegungstherapie; MET, Multifaceted Empathy Test; IRI, Interpersonal Reactivity Index; ASIM, Assessment of Spontaneous Interaction in Movement; CEEQ, Cognitive and Emotional Empathy Questionnaire; IRI, Interpersonal Reactivity Index; CCC-2, Children’s Communication Checklist, Second Edition; HIS, Heidelberger State Inventory; SRS-2, Social Responsiveness Scale, Second Edition; VABS-MB, Maladaptive Behaviour Subscale of the Vineland Adaptive Behaviour Scales; FQoL, Family Quality of Life; PPVT-4, Peabody Picture Vocabulary Test, Fourth Edition; VABS-MB, Vineland Adaptive Behavior Scales; ABC, Autistic Behavior Checklist; MOASPOS, Modified Overt Aggression Scale; ASQ, Autism Screening Questionnaire; FIM, Functional Independence Measure; PEP-3, Psychoeducational Profile, Third Edition

The total sample size included in this meta-analysis was 408 participants, including both experimental (n=214) and control (n=195) groups. Eight studies (36, 38–44) focused on participants with official ASD diagnoses based on the established diagnostic criteria, while three studies (35, 37, 45) did not provide specific details regarding the methods used for ASD diagnosis. The participants ranged from preschool children to adults. Music, dance, rhythm, and piano training were used as interventions in the experimental group, and Waitlis, social skill training, tabletop activities, traditional movement training, non-music control intervention, and traditional care were conducted in the experimental group—intervention settings, including the group and individual. The intervention frequency ranged from one to four times per week, each lasting 30–90 min. It is important to note that session time here refers to the total intervention time, not the rhythm-based intervention. The current systematic review conducted an extensive search strategy to investigate the potential significance of rhythm information for individuals diagnosed with ASD. A total of 11 studies were incorporated in the systematic review, offering valuable insights for enhancing rehabilitation programs for individuals with ASD.

### 3.3 Meta-analysis for effects of rhythm-based interventions for individuals with ASD

**Figure 2:**
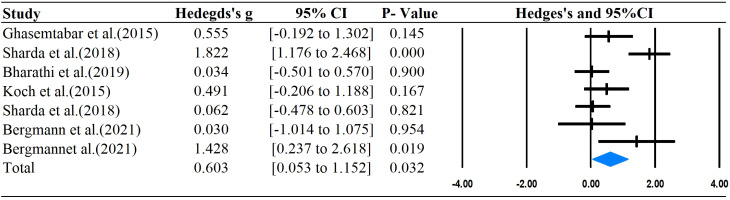
Forest plots for the effect of rhythm-based interventions on overall social skills.

**Figure 3:**
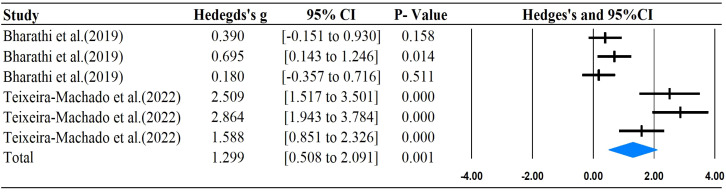
Forest plots for the effect of rhythm-based interventions on social interaction.

**Figure 4:**
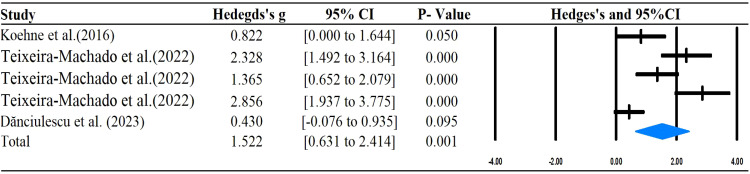
Forest plots for the effect of rhythm-based interventions on communication.

**Figure 5:**
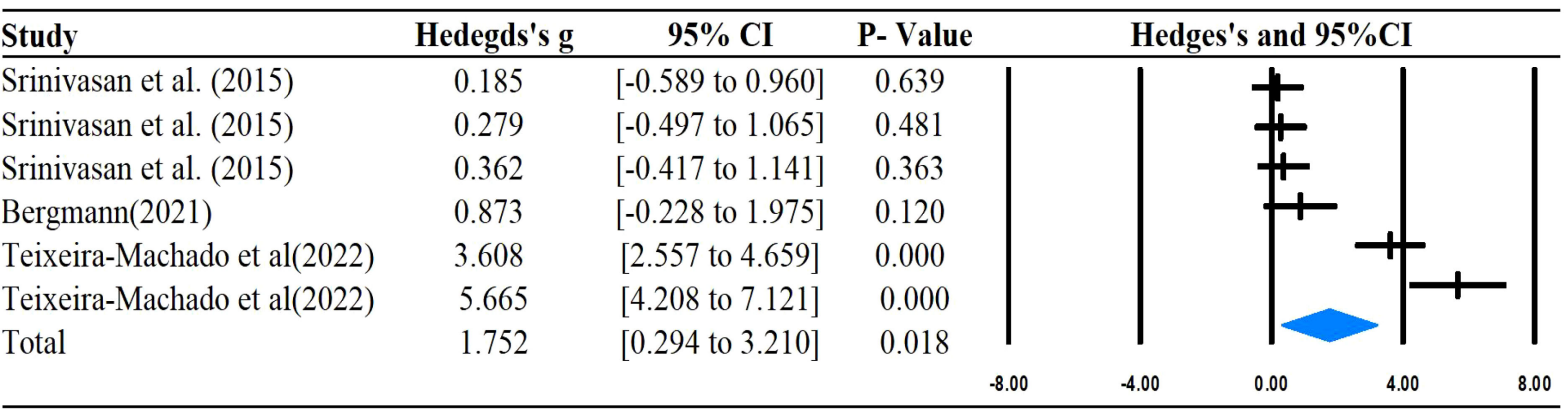
Forest plots for the effect of rhythm-based interventions on emotion.

**Figure 6:**
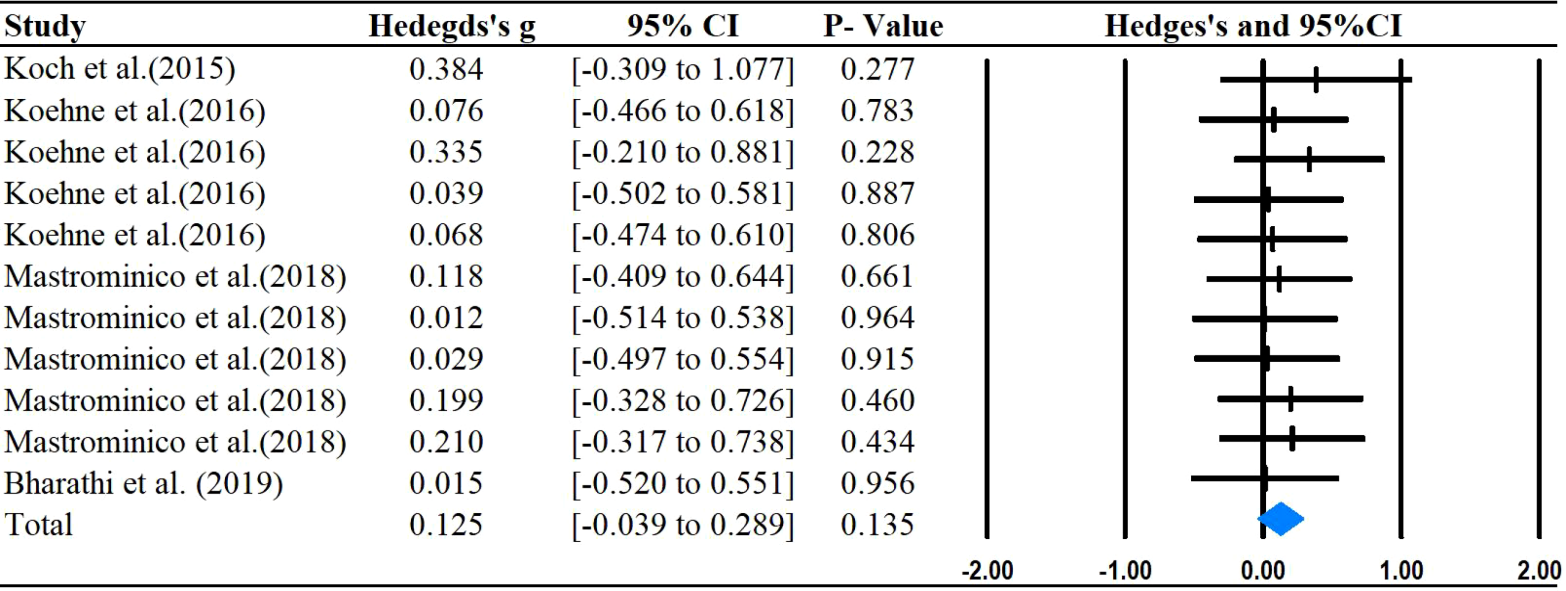
Forest plots for the effect of rhythm-based interventions on empathy.

The main outcomes of this meta-analysis are described in **Table 2** and **Figures 2**–**6**. Studies investigating the effects of rhythm-based interventions on social skills in individuals with ASD (n = 248) were identified as suitable for and included in the meta-analysis. Of the five (35, 36, 38, 39, 43) studies with eight outcomes that evaluated rhythm-based interventions on overall social skills, the results presented in **Figure 2** indicate a medium effect size for individuals with ASD on overall social skills (g= 0.681; 95%CI[0.075 to 1.286], *P*  < 0.05) with high heterogeneity. Two (36, 45) studies with six outcomes evaluated social interaction, and the results showed that rhythm-based interventions had a large effect on the social interaction of individuals with ASD (g = 1.299; 95%CI[0.508–2.091], *P*  < 0.05) with high heterogeneity. Three (37, 40, 45) studies with five outcomes revealed large effects of rhythm-based interventions on communication (g = 1.522, 95% CI [0.631 to 2.414], P  < 0.05) in tandem with high heterogeneity. Three (35, 44, 45) studies with six outcomes concentrated on emotion specified significantly large effects(g = 1.752, 95% CI [0.294–3.210], *P*  < 0.05) with high heterogeneity. In addition, four (36, 39, 40, 42) studies with 11 outcomes showed a favorable but non-significant effect (g=0.125, 95% CI [-0.039 to 0.289], *P* >  0.05) of rhythm-based interventions on empathy in individuals with ASD with low heterogeneity.

**Table 2:** Summary of the effect of the rhythm-based interventions for individuals with ASD.

Rhythm-based interventions for individuals with ASD				
**Patients**: individuals with ASD **Intervention**: experimental group (rhythm-based interventions)				
Outcomes	Hedges’s (95%CI)	z-Value	*P* Value	I ^2^, %
Overall social skills	0.681 (0.075 to 1.286)	2.203	**0.028**	78.890
Social interaction	1.299 (0.508 to 2.091)	3.218	**0.001**	88.038
Communication	1.522 (0.631 to 2.414)	3.346	**0.001**	86.383
Emotion	1.752 (0.294 to 3.210)	2.356	**0.018**	93.139
Empathy	0.125 (-0.039 to 0.289)	1.494	0.135	0.000

Crude odds ratio is significantly different.

Bold values indicate that the crude odds ratio is significantly different.

### 3.4 Quality assessment

**Table 3** provides an evaluation of the quality of the studies included in the analysis. On average, the studies exhibited a high level of quality, with a mean quality score of 7. Many of the studies received lower marks due to the absence of blinding, which is often unavoidable in exercise intervention trials given the study design. Scoring two points for the blinding of participants and therapists involved in these interventions is particularly difficult.

**Table 3:** Methodological quality assessment of included studies.

Study	Score	Methodological Quality	PEDro Item										
			1	2	3	4	5	6	7	8	9	10	11
LaGasse (2014) (41)	6	Good	**+**	**+**	**+**					**+**		**+**	**+**
Ghasemtabar et al. (2015) (38)	7	Good	**+**	**+**		**+**				**+**	**+**	**+**	**+**
Koch et al. (2015) (39)	6	Good	**+**	**+**		**+**				**+**	**+**	**+**	**+**
Srinivasan et al. (2015) (44)	7	Good	**+**	**+**		**+**				**+**	**+**	**+**	**+**
Koehne et al. (2016) (14)	8	Good	**+**	**+**		**+**	**+**			**+**	**+**	**+**	**+**
Mastrominico et al. (2018) (42)	6	Good	**+**	**+**		**+**					**+**	**+**	**+**
Sharda et al. (2018) (43)	10	excellent	**+**	**+**	**+**	**+**		**+**	**+**	**+**	**+**	**+**	**+**
Bharathi et al. (2019) (25)	7	Good	**+**	**+**		**+**				**+**	**+**	**+**	**+**
Bergmann (2021) (35)	7	Good	**+**	**+**		**+**				**+**	**+**	**+**	**+**
Teixeira-Machado et al. (2022) (45)	7	Good	**+**	**+**	**+**	**+**					**+**	**+**	**+**
Dănciulescu et al. (2023) (37)	6	Good	**+**	**+**		**+**					**+**	**+**	**+**

## 4 Discussion

This is the first systematic quantitative analysis review focusing on specific rhythm techniques as the primary component of interventions targeting social skills in individuals with ASD. Existing evidence suggests a moderate to large effect of rhythm-based interventions on overall social skills (g = 0.681), social interaction (g = 1.299), communication (g = 1.522), and emotion (g = 1.752) compared with the control group. However, we did not observe a statistically significant effect (g = 0.125) of rhythm-based interventions on empathy among individuals with ASD. This is partially consistent with an earlier meta-analysis supporting rhythm-based interventions for ASD in several social skills (21, 22). For example, Chen et al. found that dance practice can significantly improve social interaction but does not significantly affect empathy for individuals with ASD. Another meta-analysis of music therapy indicated that music therapy only improved social reactions among children with ASD. However, music therapy did not improve social adaptive behavior or speech in children with ASD.

Our findings regarding the positive effects of rhythm-based interventions on overall social skills extend the results of previous systematic reviews (4). In addition, we mentioned previously the results of dance and music therapy. Our review confirmed a moderate to large training effect on overall social skills in individuals with ASD. This provides positive information for the rehabilitation education of children with clinical ASD. The underlying mechanism of rhythm-based interventions that induced social skill improvements may be related to two aspects. First, rhythm-based interventions usually involve a clear beat on the metronome. These clear beats can easily evoke synchronous behaviors (such as emotes, pronunciation, intonation, and body expression) and neural synchronization (18, 46). These synchronous behaviors and neural synchronization have significant social implications, particularly in enhancing social connections (47, 48). Another possible explanation is that these rhythm-based interventions are usually accompanied by music to sing or dance, which increases the production of the neuropeptides oxytocin and vasopressin (49, 50). These two hormones are beneficial for improving social skills in individuals with ASD (51, 52).

A lack of communication and social interaction is regarded as one of the core characteristics of primary social dysfunction in ASD (53). Interventions that utilize rhythm have been shown to markedly enhance social interaction and communication when compared to other forms of physical activity or no intervention at all. Several previous reviews also support these results. Notice that all primal studies interventions can be considered embodied rhythmic interventions. Therapists could incorporate rhythm into interventions for individuals with ASD to improve communication, social interaction, and emotional organization (54). By using rhythm as a tool, therapists can connect with individuals with ASD on a social level, fostering a sense of understanding and recognition (11). This in turn can lead to stronger social bonds and encourage prosocial behavior among pairs of individuals with ASD (55). Additionally, research has shown that activities involving synchronization, entrainment of rhythmic vocalizations, and bimanual motor actions can effectively stimulate speech, motor skills, and language related-brain networks in individuals with ASD (8, 25). These early findings suggest that rhythm- based interventions can promote cortical reorganization and functional changes through neuroplasticity.

Empathy, which is the capacity to comprehend and resonate with the emotions of others, showed varied outcomes with rhythm-based interventions, leading to a meta-analytic effect size that was not significant. The dance intervention also showed no improvement in empathy for ASD in a recent meta-analysis (21). This may have something to do with the complexity of empathy. Empathy is a multi-dimensional phenomenon encompassing both cognitive and affective components. These two components are cognitive empathy and affective empathy (56). Our study does not distinguish between cognitive and affective empathy (56) in calculating the process. Therefore, there may be some bias. Future research should determine the effects of rhythm intervention on different empathies. Conversely, some researchers argue that the neurological systems responsible for empathy may be compromised in individuals with autism spectrum disorders, making it challenging to see improvement through short-term training (57). Thus, Future research should consider rhythm-based interventions administered at higher dosage levels and over extended periods for better efficacy.

The current systematic review conducted an extensive search strategy to investigate the potential significance of rhythm information for individuals diagnosed with ASD. In total, 11 studies were included in the systematic review, providing helpful information for optimizing rehabilitation programs in individuals with ASD. A recent study reported that individuals can use rhythm information to regulate neural oscillatory characteristics and cognitive contributions. This provides a preliminary theoretical basis for our result regarding rhythm intervention to improve social cognition (58). Compared with TMS and tACS, rhythmic visual and sound stimulation has less risk and higher acceptability and has a specific application prospect in clinical rehabilitation. Whether rhythm intervention can be used to regulate brain activity and improve social skills for individuals with ASD still needs more research to explore the neural mechanism behind it.

The present study contains some limitations that may limit the quality of the evidence. First, only studies in English were included; hence some relevant studies in other languages might have been overlooked. Second, this study had significant heterogeneity, and further subgroup analysis of the results was impossible due to the limited number of RCTs. Moreover, it should be mentioned that the current study only analyzed rhythm as a factor of intervention rather than an intervention.

## 5 Conclusion

Rhythm-based interventions are an effective way to improve the social ability of individuals with ASD. This result indicates the importance of rhythm in the clinical rehabilitation of children with ASD. We suggest adding appropriate rhythmic elements to clinical interventions, especially for individuals with ASD who are less socially competent. Future research could provide insight into the mechanisms underlying the benefits of rhythm interventions in individuals with ASD.

## Data Availability

The raw data supporting the conclusions of this article will be made available by the authors, without undue reservation.

## Supplementary material

Supplementary Table 1 PRISMA checklist

Supplementary Table 2 Search strategy

Supplementary Table 3 List of excluded studies with reason for full text
