# Supplementary material for: The benefit of rhythm-based interventions for individuals with autism spectrum disorder: a systematic review and meta-analysis with random controlled trials
Source: Front Psychiatry. 2024 Sep 27;15:1436170. doi: 10.3389/fpsyt.2024.1436170 (PMC11466867; doi:10.3389/fpsyt.2024.1436170)
Supplement: Supplementary Table 1 — PRISMA checklist [file Table1.docx]

Supplementary material

SUPPLEMENTARY TABLE 1

PRISMA checklist

SUPPLEMENTARY TABLE 2

Search strategy

SUPPLEMENTARY TABLE 3

List of excluded studies with reason for full text

Table1. PRISMA checklist

| Section and Topic | Item # | Checklist item | Location where item is reported |
| --- | --- | --- | --- |
| TITLE | | |  |
| Title | 1 | Identify the report as a systematic review. | Title |
| ABSTRACT | | |  |
| Abstract | 2 | See the PRISMA 2020 for Abstracts checklist. | Abstract |
| INTRODUCTION | | |  |
| Rationale | 3 | Describe the rationale for the review in the context of existing knowledge. | 1.Introduction |
| Objectives | 4 | Provide an explicit statement of the objective(s) or question(s) the review addresses. | 1.Introduction s |
| METHODS | | |  |
| Eligibility criteria | 5 | Specify the inclusion and exclusion criteria for the review and how studies were grouped for the syntheses. | 2.2 Eligibility Criteria |
| Information sources | 6 | Specify all databases, registers, websites, organisations, reference lists and other sources searched or consulted to identify studies. Specify the date when each source was last searched or consulted. | 2.1 Search Strategy |
| Search strategy | 7 | Present the full search strategies for all databases, registers and websites, including any filters and limits used. | Supplementary etable 2 Search Strategy |
| Selection process | 8 | Specify the methods used to decide whether a study met the inclusion criteria of the review, including how many reviewers screened each record and each report retrieved, whether they worked independently, and if applicable, details of automation tools used in the process. | 3.1 Study identification |
| Data collection process | 9 | Specify the methods used to collect data from reports, including how many reviewers collected data from each report, whether they worked independently, any processes for obtaining or confirming data from study investigators, and if applicable, details of automation tools used in the process. | 2.3 Study Selection and Data Extraction |
| Data items | 10a | List and define all outcomes for which data were sought. Specify whether all results that were compatible with each outcome domain in each study were sought (e.g. for all measures, time points, analyses), and if not, the methods used to decide which results to collect. | 2.3 Study Selection and Data Extraction |
|  | 10b | List and define all other variables for which data were sought (e.g. participant and intervention characteristics, funding sources). Describe any assumptions made about any missing or unclear information. | 3.2 Study characteristics |
| Study risk of bias assessment | 11 | Specify the methods used to assess risk of bias in the included studies, including details of the tool(s) used, how many reviewers assessed each study and whether they worked independently, and if applicable, details of automation tools used in the process. | 2.4 Quality Assessment |
| Effect measures | 12 | Specify for each outcome the effect measure(s) (e.g. risk ratio, mean difference) used in the synthesis or presentation of results. | 2.5 Data Analysis |
| Synthesis methods | 13a | Describe the processes used to decide which studies were eligible for each synthesis (e.g. tabulating the study intervention characteristics and comparing against the planned groups for each synthesis (item #5)). | 2.5 Data Analysis |
|  | 13b | Describe any methods required to prepare the data for presentation or synthesis, such as handling of missing summary statistics, or data conversions. | 2.5 Data Analysis |
|  | 13c | Describe any methods used to tabulate or visually display results of individual studies and syntheses. | 2.5 Data Analysis |
|  | 13d | Describe any methods used to synthesize results and provide a rationale for the choice(s). If meta-analysis was performed, describe the model(s), method(s) to identify the presence and extent of statistical heterogeneity, and software package(s) used. | 2.5 Data Analysis |
|  | 13e | Describe any methods used to explore possible causes of heterogeneity among study results (e.g. subgroup analysis, meta-regression). | 2.5 Data Analysis |
|  | 13f | Describe any sensitivity analyses conducted to assess robustness of the synthesized results. | 2.5 Data Analysis |
| Reporting bias assessment | 14 | Describe any methods used to assess risk of bias due to missing results in a synthesis (arising from reporting biases). | 2.5 Data Analysis |
| Certainty assessment | 15 | Describe any methods used to assess certainty (or confidence) in the body of evidence for an outcome. | NA |
| RESULTS | | |  |
| Study selection | 16a | Describe the results of the search and selection process, from the number of records identified in the search to the number of studies included in the review, ideally using a flow diagram. | 3.1 Study identification |
|  | 16b | Cite studies that might appear to meet the inclusion criteria, but which were excluded, and explain why they were excluded. | Supplementary eTable3. List of excluded studies with reason for full text |
| Study characteristics | 17 | Cite each included study and present its characteristics. | 3.2 Study characteristics |
| Risk of bias in studies | 18 | Present assessments of risk of bias for each included study. | 3.4 Quality assessment |
| Results of individual studies | 19 | For all outcomes, present, for each study: (a) summary statistics for each group (where appropriate) and (b) an effect estimate and its precision (e.g. confidence/credible interval), ideally using structured tables or plots. | 3.3 Meta-analysis for effects of rhythm-based interventions for individuals with ASD |
| Results of syntheses | 20a | For each synthesis, briefly summarise the characteristics and risk of bias among contributing studies. | 3.2 Study characteristics |
|  | 20b | Present results of all statistical syntheses conducted. If meta-analysis was done, present for each the summary estimate and its precision (e.g. confidence/credible interval) and measures of statistical heterogeneity. If comparing groups, describe the direction of the effect. | 3.3 Meta-analysis for effects of rhythm-based interventions for individuals with ASD |
|  | 20c | Present results of all investigations of possible causes of heterogeneity among study results. | 3.3 Meta-analysis for effects of rhythm-based interventions for individuals with ASD |
|  | 20d | Present results of all sensitivity analyses conducted to assess the robustness of the synthesized results. | NA |
| Reporting biases | 21 | Present assessments of risk of bias due to missing results (arising from reporting biases) for each synthesis assessed. | 3.4 Quality assessment |
| Certainty of evidence | 22 | Present assessments of certainty (or confidence) in the body of evidence for each outcome assessed. | NA |
| DISCUSSION | | |  |
| Discussion | 23a | Provide a general interpretation of the results in the context of other evidence. | 1. Discussion |
|  | 23b | Discuss any limitations of the evidence included in the review. | 4.Discussion |
|  | 23c | Discuss any limitations of the review processes used. | 4.Discussion |
|  | 23d | Discuss implications of the results for practice, policy, and future research. | 4.Discussion |
| OTHER INFORMATION | | |  |
| Registration and protocol | 24a | Provide registration information for the review, including register name and registration number, or state that the review was not registered. | 2. Methods |
|  | 24b | Indicate where the review protocol can be accessed, or state that a protocol was not prepared. | 2. Methods |
|  | 24c | Describe and explain any amendments to information provided at registration or in the protocol. | NA |
| Support | 25 | Describe sources of financial or non-financial support for the review, and the role of the funders or sponsors in the review. | NA |
| Competing interests | 26 | Declare any competing interests of review authors. | Conflict of Interest Statement |
| Availability of data, code and other materials | 27 | Report which of the following are publicly available and where they can be found: template data collection forms; data extracted from included studies; data used for all analyses; analytic code; any other materials used in the review. | NA |

eTable 2. Search Strategy

| Database | Detail of Search Strategy |
| --- | --- |
| PubMed(ti, ab, kw)  Embase(ti, ab, kw)  PsychInfo(ti, ab)  Scoups(ti, ab, kw) | (music OR sing OR song OR “sound therapy” OR dance OR dancing  OR gymnastics OR eurhythmics OR “body psychotherapy” OR drum OR drumming OR rhythmic OR metronome  OR tempo OR rhythm) AND (“autism spectrum disorders” OR autistic OR autism OR “pervasive developmental disorder” OR “autistic disorder”) |

eTable3. List of excluded studies with reason for full text

| Study | Reason |
| --- | --- |
| Balasubramanian V, Bharathi G, Vellinigri B. Development of social interaction skills of autistic children’s by musical therapy[J]. Journal of the Neurological Sciences, 2019, 405: 49. | Conference |
| Cibrian F L, Madrigal M, Avelais M, et al. Supporting coordination of children with ASD using neurological music therapy: A pilot randomized control trial comparing an elastic touch-display with tambourines[J]. Research in developmental disabilities, 2020, 106: 103741. | Rhythmic intervention was performed in both experimental group and control group |
| Crawford M J, Gold C, Odell-Miller H, et al. International multicentre randomised controlled trial of improvisational music therapy for children with autism spectrum disorder: TIME-A study[J]. Health Technology Assessment, 2017, 21(59): 1-40. | Data was not available |
| Gattino G S, Riesgo R S, Longo D, et al. Effects of relational music therapy on communication of children with autism: a randomized controlled study[J]. Nordic Journal of Music Therapy, 2011, 20(2): 142-154. | Conference |
| He R. The Intervention of Music Therapy on Behavioral Training of High-Functioning Autistic Children under Intelligent Health Monitoring[J]. Applied Bionics and Biomechanics, 2022, 2022. | Conference |
| Rabeyron T, Del Canto J P R, Carasco E, et al. A randomized controlled trial of 25 sessions comparing music therapy and music listening for children with autism spectrum disorder[J]. Psychiatry research, 2020, 293: 113377. | Non-rhythm-based interventions in experimental group. |
| Souza-Santos C, dos Santos J F, Azevedo-Santos I, et al. Dance and equine-assisted therapy in autism spectrum disorder: Crossover randomized clinical trial[J]. Clinical Neuropsychiatry, 2018, 15(5). | Non-rhythm-based interventions in experimental group. |
| Yoo G E, Kim S J. Dyadic drum playing and social skills: Implications for rhythm-mediated intervention for children with autism spectrum disorder[J]. Journal of music therapy, 2018, 55(3): 340-375. | Non-randomized control |
| Yurteri N, Akdemir M. The effect of music therapy on autistic symptoms and quality of life in children with autism spectrum disorder/Otizm spektrum bozuklugu olan cocuklarda muzik terapinin otizm belirtileri ve yasam kalitesine etkisi[J]. Anadolu Psikiyatri Dergisi, 2019, 20(4): 436-442. | Full text not available |
| Arzoglou D, Tsimaras V, Kotsikas G, et al. The effect of [alpha] tradinional dance training program on neuromuscular coordination of individuals with autism[J]. Journal of Physical Education and Sport, 2013, 13(4): 563. | Results related to non-socialskills |
| Bieleninik Ł, Geretsegger M, Mössler K, et al. Effects of improvisational music therapy vs enhanced standard care on symptom severity among children with autism spectrum disorder: The TIME-A randomized clinical trial[J]. Jama, 2017, 318(6): 525-535. | Correction |
| Chomoriti K, Lykesas G, Kapodistria L, et al. THE EFFECTIVENESS OF A TRADITIONAL DANCE PROGRAM ON BALANCE OF PRIMARY EDUCATION STUDENTS WITH AUTISM SPECTRUM DISORDER[J]. Age (years), 2021, 11(2.26): 12-1.41. | Results related to non-socialskills |
| El-Tellawy M M, Ahmad A R, Saad K, et al. Effect of hyperbaric oxygen therapy and tomatis sound therapy in children with autism spectrum disorder[J]. Progress in Neuro-Psychopharmacology and Biological Psychiatry, 2022, 113: 110457. | Non-rhythm-based interventions in experimental group. |
| Lim H A, Draper E. The effects of music therapy incorporated with applied behavior analysis verbal behavior approach for children with autism spectrum disorders[J]. Journal of music therapy, 2011, 48(4): 532-550. | Results related to non-socialskills |
| Lundqvist L O, Andersson G, Viding J. Effects of vibroacoustic music on challenging behaviors in individuals with autism and developmental disabilities[J]. Research in Autism Spectrum Disorders, 2009, 3(2): 390-400. | Non-rhythm-based interventions in experimental group. |
| MacDonald-Prégent A, Saiyed F, Hyde K, et al. Response to Music-Mediated Intervention in Autistic Children with Limited Spoken Language Ability[J]. Journal of autism and developmental disorders, 2023: 1-15. | Results related to non-socialskills |
| Schwartzberg E T, Silverman M J. Effects of music-based social stories on comprehension and generalization of social skills in children with autism spectrum disorders: A randomized effectiveness study[J]. The Arts in Psychotherapy, 2013, 40(3): 331-337. | Non-rhythm-based interventions in experimental group. |
| Srinivasan S M, Kaur M, Park I K, et al. The effects of rhythm and robotic interventions on the imitation/praxis, interpersonal synchrony, and motor performance of children with autism spectrum disorder (ASD): a pilot randomized controlled trial[J]. Autism research and treatment, 2015, 2015. | Results related to non-socialskills |
| Cahart M S, Amad A, Draper S B, et al. The effect of learning to drum on behavior and brain function in autistic adolescents[J]. Proceedings of the National Academy of Sciences, 2022, 119(23): e2106244119. | Full text not available |
| Moradi H, Sohrabi M, Taheri H, et al. The effects of different combinations of perceptual-motor exercises, music, and vitamin D supplementation on the nerve growth factor in children with high-functioning autism[J]. Complementary Therapies in Clinical Practice, 2018, 31: 139-145. | Results related to non-socialskills |
| Ren H, Ren G, Zhan Y, et al. Examining the efficacy of dance movement and music mixed treatment on social communication impairment in children with autism—Based on family parent-child situation[J]. Frontiers in Psychology, 2022, 13: 937564. | Results related to non-socialskills |
| Srinivasan S M, Eigsti I M, Gifford T, et al. The effects of embodied rhythm and robotic interventions on the spontaneous and responsive verbal communication skills of children with Autism Spectrum Disorder (ASD): A further outcome of a pilot randomized controlled trial[J]. Research in autism spectrum disorders, 2016, 27: 73-87. | Results related to non-socialskills |
